# Nigral Iron Elevation Is an Invariable Feature of Parkinson's Disease and Is a Sufficient Cause of Neurodegeneration

**DOI:** 10.1155/2014/581256

**Published:** 2014-01-16

**Authors:** Scott Ayton, Peng Lei

**Affiliations:** The Florey Institute of Neuroscience and Mental Health, University of Melbourne, Kenneth Myer Building at Genetics Lane on Royal Parade, Parkville, VIC 3010, Australia

## Abstract

Parkinson's disease (PD) is a neurodegenerative disorder characterized by motor deficits accompanying degeneration of substantia nigra pars compactor (SNc) neurons. Although familial forms of the disease exist, the cause of sporadic PD is unknown. Symptomatic treatments are available for PD, but there are no disease modifying therapies. While the neurodegenerative processes in PD may be multifactorial, this paper will review the evidence that prooxidant iron elevation in the SNc is an invariable feature of sporadic and familial PD forms, participates in the disease mechanism, and presents as a tractable target for a disease modifying therapy.

## 1. Introduction

The substantia nigra pars compactor (SNc) degenerates in Parkinson's disease (PD), which precipitates motor disabilities such as tremor and bradykinesia that characterize the disease. Symptomatic therapy, such as Levodopa, restores dopamine levels but is ineffectual in altering the progression of the disease. While multiple brain regions are decorated by Lewy bodies (the defining pathological feature of PD) [1], it is unknown why the SNc is selectively vulnerable to neurodegeneration. Prooxidant iron accumulation in this nucleus is one possible reason. The SNc is particularly rich in iron [2], which increases with age [3]. PD is complicated by exaggerated iron retention in this nucleus, which was a historically early finding [4], and has since been repeatedly observed using various quantifiable techniques (Table 1). It is proposed that this high basal iron content, with the vulnerability to accumulate iron with age and in disease, makes this region susceptible to PD neurodegeneration. However, a limitation of postmortem tissue analysis is that, most likely, the tissues analyzed were obtained from end-stage patients, and from this evidence alone it is not possible to claim if iron is pathogenic in the disease or if it is an epiphenomenon.

## 2. Iron Accumulation in Parkinson's Disease: Cause or Effect?

Is iron elevation contributing to neuronal death in Parkinson's disease, or is it simply a feature of dying neurons? To address this important question, this paper will review three separate lines of evidence: (1) *in vivo* iron-imaging technology, (2) rare genetic disorders of iron metabolism, and (3) animal models.

### 2.1. *In Vivo* Iron-Imaging Technology

The iron hypothesis of Parkinson's disease has been revitalized by the ability to visualize and quantify iron elevation in the SNc of living patients, first by a technique called transcranial sonography (TCS) then later by T_2_*-weighted MRI. TCS has been employed for a number of years to quantify the echogenicity of the SN in PD patients. Echogenicity refers to the ability of a substance to return the sound wave back to the receiver. Tissue laden with iron has greater echogenicity allowing iron elevation in PD to be visualized with this technique [5–11]. TCS is a promising diagnostic tool for PD with positive predictive value of 85.7% and a negative predictive value of 82.9% [10]. TCS has been suggested as the earliest predictive test for PD (with the exception of genetic testing in rare familial cases) [12]. Increased echogenicity in otherwise healthy individuals has been associated with reduced [18F]-dopa uptake in the striatum [13] and minor motor abnormalities in older patients [14] possibly suggesting early, presymptomatic degeneration. Tellingly, nonsymptomatic individuals with increased echogenicity have 17 times the risk of acquiring PD after 3 years when compared to individuals with a normal echogenetic profile [15]. The early elevation of iron identified using *in vivo* imaging techniques could position this phenomenon upstream of neurodegeneration in the PD mechanism.

Increased iron in the SN has also been determined by quantifying relaxation times of T_2_- and T_2_*-weighted MRI, which strongly correlates with tissue iron levels [16]. Iron elevation by MRI has been correlated with disease severity [17, 18] and duration [19]. In a 3-year-followup study, iron measured by T_2_* MRI was shown to progressively increase in PD subjects but not controls [20]. It also has been implicated in diagnosis of PD with reported sensitivity of 100% and specificity of 80% in a small cohort [21].

Iron elevation is also a common feature of familial PD. PD-associated Park genes encoding proteins, alpha synuclein, leucine-rich repeat kinase 2 (LRRK2), PTEN-induced putative kinase 1 (PINK1), Parkin, and DJ-1, are associated with iron accumulation in the SN as measured by TCS, demonstrating that iron elevation is an invariable feature of multiple PD modalities (Table 2).

Imaging technology has allowed visualization of iron elevation as (a) a risk factor for PD, (b) an early event in the progression of the disease, (c) a feature that correlates with disease severity and duration, and (d) a feature of all causes of PD (measured so far). But imaging technology has not afforded us direct evidence to conclude if iron elevation is epiphenomenal or part of the disease process.

### 2.2. Rare Genetic Disorders of Brain Iron Metabolism Often Present as PD

Evidence placing iron as a mediating factor of PD neurodegeneration is drawn from rare genetic disorders that are known to interfere with the iron-handling pathway. The following examples demonstrate that a primary elevation in nigral iron is a sufficient cause of Parkinsonian neurodegeneration; thus iron elevation evidenced in familial and sporadic cases of PD has the clear potential to contribute to the degenerative process.

#### 2.2.1. Aceruloplasminemia

Ceruloplasmin is multicopper oxidase that converts ferrous to ferric iron and is required for the export of iron from cells [22]. Aceruloplasminemia is a rare genetic disorder of ceruloplasmin dysfunction that results in an iron retention within various tissues [23, 24] that can be recapitulated in ceruloplasmin knockout mice [25–27]. Dozens of ceruloplasmin mutations have been identified and some induce Parkinsonism in affected individuals [28]. In particular, three missense mutations in ceruloplasmin- I63T, D554E, and R793H, have been shown to exhibit reduced ferroxidase activity in the plasma of patients [29], increased nigral iron content, and a Parkinsonian presentation in affected individuals [30].

#### 2.2.2. Neurodegeneration with Brain Iron Accumulation Type 1

Neurodegeneration with brain iron accumulation type 1 (NBIA1) is caused by a mutation in pantothenate kinase 2 (PANK2). PANK2 catalyses the initial step in coenzyme A synthesis and mutations have reduced catalytic activity [31] and basal ganglia iron accumulation [32]. Patients with this mutation often present with Parkinsonism [33]. Brains of affected patients contain the Parkinson's Lewy body pathology [34–36], possibly suggesting that iron accumulation is upstream of alpha synuclein deposition in idiopathic PD.

#### 2.2.3. Neuroferritinopathy

Neuroferritinopathy, also called neurodegeneration with brain iron accumulation type 2, is a rare autosomal dominant neurodegenerative disease caused by one of seven known mutations to the ferritin light chain protein [37–43]. The mutation causes increased iron and ferritin deposition in the brain but not other organs. Iron elevation in the brain results in various extrapyramidal motor symptoms including dystonia, chorea, parkinsonism, and tremor.

### 2.3. Animal Models

A variety of animal models have demonstrated that iron elevation is sufficient to cause neurodegeneration in the nigra. Direct injection of iron into the midbrain region of rats causes SN neuronal loss [44]. A number of studies employing an iron feeding protocol to neonatal mice have shown Parkinsonism and nigral degeneration in these mice when they reach adulthood [45–47]. These studies take advantage of the immature blood brain barrier which allows elevated systemic iron to permeate the brain. Neonatal iron fed mice are also more susceptible to 1-methyl-4-phenyl-1,2,3,6-tetrahydropyridine (MPTP) intoxication [48].

Toxin models of PD, MPTP, and 6-hydroxydopamine (6-OHDA) also recapitulate iron elevation within the SN [49, 50]. The changes in iron within the SN cells are complex and poorly understood. Within the first 2–4 days after treatment of MPTP, there is an increase in chelatable iron within the mitochondria [51], preceding total iron elevation, 1-2 weeks after administration. Iron chelator drugs are effective in attenuating the damage caused by these Parkinsonian toxins [52, 53] demonstrating that iron is a mediator of neurodegeneration in toxin models of PD and that iron chelation is an attractive therapeutic option for PD.

## 3. The Consequences of Iron Elevation: Implications for the Disease Mechanism

### 3.1. Oxidative Stress

Iron elevation can cause oxidative stress-mediated cell death. Within biological systems iron can react with oxygen to catalyze the formation of the toxic hydroxyl radical via the Fenton reaction. This reaction is dependent on the ability of iron to alter its valence state between ferrous (Fe^2+^) and ferric (Fe^3+^) species [54]. Inappropriate iron retention in PD could promote oxidative stress within this tissue. Post mortem PD-affected brains display increased lipid peroxidation [55], oxidative damage to DNA [56], and lowered levels of the reduced form of glutathione [57], which all reflect oxidative stress.

### 3.2. Alpha Synuclein Deposition

Alpha synuclein is often considered the Parkinson's protein owning to extensive links to the disease. Three genetic mutations of alpha synuclein, A53T [58], A30P [59], and E46K [60], along with gene duplication [61] or triplication [62] have been associated with inheritable forms of PD. Aggregated alpha synuclein is also the major component of Lewy bodies, the pathological hallmark for PD [63]. Alpha synuclein deposition could be contributed to by iron exposure. Alpha synuclein binds to iron [64–66], which accelerates its aggregation into fibrils [67, 68]. Alpha synuclein has also been shown to directly generate hydrogen peroxide when it aggregates and, in the presence of iron, produce toxic hydroxyl radicals [69]. In cultured neurons, iron has been shown to cause aggregation of alpha synuclein [70–73]. Collectively these findings demonstrate that iron can theoretically impact the aggregation of alpha synuclein. Iron is also found enriched in Lewy bodies [74], providing *in vivo* evidence that iron elevation could induce Lewy body deposition in PD.

## 4. Mechanisms of Iron Accumulation in PD

Understanding how iron accumulates in PD may provide opportunities to target this process pharmacologically. There is only a weak association between environmental iron exposure and development of PD [75, 76], suggesting that endogenous factors or metabolic dysregulation is the genesis for elevated iron in PD. Peripheral iron status, modified by diet or various genetic disorders (e.g., hemochromatosis), is rarely reflected by concomitant changes to iron in the brain. While peripheral iron has access to the brain [77] and iron consumed in the diet is distributed to the brain in comparable levels to other organs [78], the brain retains mostly stable throughout life [79–81]. The brain is able to control iron levels in a narrow range by recruiting various protein machinery. While many reviews discuss the iron regulatory pathways of the brain [82, 83], this paper will discuss the evidence that fatigue to these machinery results in iron accumulation in PD.

Various iron associated proteins have been investigated for their potential to contribute to iron accumulation in PD. The earliest such study surveyed the iron storage protein ferritin in post mortem PD brains, demonstrating a decrease in this protein compared to controls [84]. Increased iron with a concomitant decrease in ferritin within the SN could suggest an increase in the labile iron pool, making iron more available for toxic interactions. Ferritin elevation, therefore, might be neuroprotective in the disease. This was demonstrated in the MPTP model, where ferritin overexpressing mice were protected against the toxin [52].

While changes in ferritin could relate to cellular buffering capacity of iron, this does not easily explain why iron accumulates in PD. Iron elevation in PD could be contributed to by increased cellular iron uptake via transferrin receptor 1 (TfR1). However, the levels of this protein were shown not to be altered in PD (when loss of neurons was accounted for) [85], nor in the 6-OHDA model [86], arguing against a role for this protein in iron accumulation of PD. Within the periphery, the transferrin receptor is inhibited by the hemochromatosis protein as a normal component of the iron homeostatic mechanism. However, loss of function genetic mutations in the hemochromatosis protein causes pathological iron retention in peripheral tissues in hereditary hemochromatosis. The iron overload associated with hemochromatosis in peripheral tissue is also not convincingly linked to PD. The C282Y mutation in hemochromatosis protein was initially shown to increase the risk of PD and Parkinsonism [87] and this was supported by a later study [88]. However further investigations have failed to find an association with C282Y mutation and PD [89–91], although one study found two single nucleotide polymorphisms (K92N and I217T) in a single PD patient and not in controls [89]. It remains unclear what role hemochromatosis protein plays in either genetic or idiopathic PD; further it is not known if individuals with hereditary hemochromatosis have brain iron accumulation. It is likely that the peripheral hemochromatosis hormone plays a minor role in brain iron homeostasis; indeed staining for the hemochromatosis protein in brain reveals only scattered staining for the protein, localized to parts of cortex, cerebellum, and brain endothelium [92].

Another iron importing protein, transferrin receptor 2 (TfR2) was found to be increased in the rotenone model of PD [93]. It is proposed in the paper by Mastroberardino et al. [93] that TfR2 signals for iron to be deposited in the mitochondria leading to iron accumulation and oxidative damage within the SN. The function consequence of this process is unclear and TfR2 has yet to be explored in human PD tissue.

Both TfR1 and TfR2 receive iron from the transferrin protein. The complex then undergoes endocytosis where iron is transported across the endosome by the divalent metal ion transporter (DMT1). An isoform of DMT1 with an iron responsive element (DMT1 + IRE) was shown to be increased in cultured cells exposed to 6-OHDA [94] and early after MPTP intoxication in mice [95], which was later replicated [96]. Salazar et al. [96] additionally used mice with a loss of function mutation of DMT1 and showed that this was protective against MPTP induced iron elevation and toxicity. Importantly, Salazar et al. [96] also showed that DMT1+IRE was elevated in the SN of PD patients, which implicates overexpression of this protein in iron accumulation in PD.

Iron elevation in PD could also be contributed by failure of iron export. Ferroportin is the only known iron exporting channel in mammals [97]. Ferroportin requires cooperation with a ferroxidase that converts intracellular ferrous iron (Fe^2+^) to ferric iron (Fe^3+^) so that extracellular ferric-binding transferrin protein can remove iron from ferroportin [98]. Ceruloplasmin is the best characterized ferroxidase protein and has been investigated as a possible contributor to iron elevation in PD. Decreased ceruloplasmin ferroxidase activity has been observed in PD cerebrospinal fluid (CSF) [99–101] and serum [102–107], while low serum ceruloplasmin activity is correlated with earlier age of PD onset [103, 105, 106]. Loss of peripheral ceruloplasmin function could therefore impact brain iron levels, and indeed peripheral and brain-derived pools of ceruloplasmin are in exchange [26]. The level of ceruloplasmin in the PD SN is unaltered in the disease [108], but its activity is selectively reduced in the SNc [26]. This is important since loss of ceruloplasmin function, as in aceruloplasminemia, often causes Parkinsonism in affected individuals [28].

Iron export could also be impacted by the tau protein. Tau protein is famously linked with Alzheimer's disease and frontotemporal dementia, but tau protein has been implicated in PD from genetic, pathological, and biochemical perspectives [109]. Tau levels are reduced in the substantia nigra in PD, which obtunds amyloid precursor protein (APP) mediated iron export [110]. APP binds to ferroportin to facilitate iron export [111], and loss of tau prevents APP trafficking to the neuronal surface to perform this function [110]. Tau knockout mice develop iron-mediated Parkinsonism [110], highlighting the neurotoxic potential for a disturbance to this pathway.

## 5. Conclusions and Therapeutic Implications 

So is iron elevation the cause of PD? Quite simply the answer has to be no, since there has to be some other process that causes iron to elevate in the first place. The same could be said of any other factor implicated in PD. If alpha synuclein is the cause of Parkinson's disease, what causes the protein to aggregate? Understanding the cause of PD may indeed be impossible to define. Even if this was understood, the factor(s) inducing the degenerative process in PD may not be propagating disease progression by the time the patient initially presents with symptoms. The degenerative processes in PD are likely to involve multiple pathways; the goal of our field should be to identify components of the disease mechanism that can be targeted pharmacologically. Iron is one such component. Iron is elevated invariably in the SNc of PD-effected brains, early in the disease processes, and brain iron overload is sufficient to cause Parkinsonism in rare genetic cases or brain iron overload, or in various animal models of PD.

Iron is also a tractable target for pharmacotherapy. Numerous iron chelators are currently in use for peripheral disorders of iron overload [112]. Iron chelator, Deferiprone, was recently shown to benefit PD patients in a phase II clinical trial (PMID: 24251381) [113]. This is the first drug to show a disease-modifying effect for PD, which highlights the potential for targeting iron for PD pharmacotherapy, and strongly implicates iron in the disease mechanism. A limitation of the iron chelators presently in the market is that they were developed to treat peripheral disorders of iron metabolism [114]. Iron chelators that have greater access to the brain through BBB are currently in development [52, 53, 110, 115].

There is also opportunity to target cellular mechanisms that cause iron to be elevated in PD. Preventing elevation of DMT1, or restoring the loss of tau or ceruloplasmin, might be additional ways to target the iron lesion. While strategies that prevent the rise in iron or remove excess iron in the disease might not be silver bullets that halt neurodegeneration altogether, the evidence outlined in this review suggests that these approaches might slow the degenerative process and therefore are attractive options for the first disease-modifying therapy for PD.

## Figures and Tables

**Table 1:** Reports of quantifiable iron in PD SN.

Iron measurement technique	Fe in PD SN (% control)	Reference
ICP-MS	135	[116]
SP	176	[117]
SP	177	[118]
AAS	107	[119]
ICP-MS	133	[120]
ICP-MS	130	[121]
X-Ray Microprobe analysis	340	[122]
SP	150	[123]
Laser microprobe analysis	145	[124]
ICPMS	156	[125]
Colorimetry	82	[126]
X-Ray absorption fine structure	201	[127]
Electron probe X-Ray microanalysis coupled with cathodoluminescence spectroscopy	200	[128]
AAS	144	[96]
X-Ray fluorescence	155	[129]
ICP-MS	140	[110]
AAS	139	[26]

Average	159.5	

ICPMS: inductively coupled plasma mass spectrometry; SP: spectrophotometry; AAS: atomic absorption spectrometry.

**Table 2:** Iron elevation (TCS) in the genetics of PD.

Locus	Gene	Mutation	Comments	Iron elevation	Reference
PARK1/4	Alpha synuclein	3 point mutations, duplication, triplication	Onset age 40–50 Rapid progression, frequent dementia. Lewy bodies present	Yes	[130]
PARK2	Parkin	>100 mutations	Age of onset 17–24 years Parkinsonism Other motor symptoms but no dementia. Lewy bodies present	Yes	[130, 131]
PARK6	PINK1	>20 mutations	Onset age 30–50 Clinical features resemble Parkin mutations. Lewy bodies present	Yes	[130, 132]
PARK7	DJ-1	3 point mutations	Onset age 20–40 Clinical features resemble Parkin mutations. Unknown if Lewy bodies are present	Yes	[130]
PARK8	LRRK2	6 point mutations	Age of onset 50s Resembles idiopathic PD. Lewy bodies present	Yes	[130, 133]
PARK9	ATP13A2	1 mutation	Age of onset 10–30 years Parkinsonism, supranuclear gaze paresis, pyramidal signs, and dementia. Unknown if Lewy bodies are present	??	
PARK14	PLA2G6	2 mutations	Age of onset 20–40 Parkinsonism Dementia, psychosis, dystonia, and hyperreflexia. Unknown if Lewy bodies are present	??	
PARK15	FBXO7	3 mutations	Age of onset 7–22 Parkinsonism, hyperreflexia, and spasticity. Unknown if Lewy bodies are present	??	

Iron accumulation [130–133] and Lewy bodies [134].
